# Surviving Burn Injury: Drivers of Length of Hospital Stay

**DOI:** 10.3390/ijerph18020761

**Published:** 2021-01-18

**Authors:** Chimdimma Noelyn Onah, Richard Allmendinger, Julia Handl, Ken W. Dunn

**Affiliations:** 1 Alliance Manchester Business School, University of Manchester, Manchester M13 9PL, UK; Richard.allmendinger@manchester.ac.uk (R.A.); Julia.handl@manchester.ac.uk (J.H.); 2 Department of Burns and Plastic Surgery, University Hospital South Manchester, Manchester M23 9LT, UK; ken.dunn@mft.nhs.uk

**Keywords:** burn care, length of stay, mental state, socioeconomic status, clustering, predictive models, regression analysis, collaborative decision-making

## Abstract

With a reduction in the mortality rate of burn patients, length of stay (LOS) has been increasingly adopted as an outcome measure. Some studies have attempted to identify factors that explain a burn patient’s LOS. However, few have investigated the association between LOS and a patient’s mental and socioeconomic status. There is anecdotal evidence for links between these factors; uncovering these will aid in better addressing the specific physical and emotional needs of burn patients and facilitate the planning of scarce hospital resources. Here, we employ machine learning (clustering) and statistical models (regression) to investigate whether segmentation by socioeconomic/mental status can improve the performance and interpretability of an upstream predictive model, relative to a unitary model. Although we found no significant difference in the unitary model’s performance and the segment-specific models, the interpretation of the segment-specific models reveals a reduced impact of burn severity in LOS prediction with increasing adverse socioeconomic and mental status. Furthermore, the socioeconomic segments’ models highlight an increased influence of living circumstances and source of injury on LOS. These findings suggest that in addition to ensuring that patients’ physical needs are met, management of their mental status is crucial for delivering an effective care plan.

## 1. Introduction

Globally, burn injury is one of the top ten causes of trauma and a leading contributor to the years of life lost from premature death and years lived in less than full health [1]. Burn patients represent one of the most complex types of patient groups due to [2] (i) the trauma of the injury; (ii) the prevalence of pre-existing conditions and (iii) the occurrence of complications during the hospital stay. The high incidence and complex aetiology of burn injuries necessitate constant evaluation for better care management, multidisciplinary intervention approaches, effective costing, funding, and prevention programs.

To better understand and manage burn patients, it is therefore vital to understand the factors that influence the recovery of patients. Past studies have identified factors such as total burn surface area (TBSA), age, presence of inhalation injury, comorbidities, complications and, in some cases, the patient’s gender as estimators of the length of stay (LOS) [3,4,5,6,7,8]. A well-developed prediction model of LOS on admission would aid in understanding burn patients better through the identification and management of significant predictors. The predictive model would also support patient triaging, the development of personalised care pathways, resource planning, and patient, family and friends counselling on the expected date of discharge [5,9]. For the model to be fully employable, it needs collaboration with the relevant stakeholders (in this case, burn care specialists) to ensure the use of relevant patient cases and factors and, subsequently, the production of an explainable model.

This paper focuses on the effect of an adverse mental state and a patient’s social environment, represented by their deprivation score—a measure of poverty—on an adult patient’s LOS. Our central hypothesis is that recovery from a burn injury is not predominantly determined by burn injury severity but is also associated with a patient’s state of mind (mental health) as well as their social environment, as captured by socioeconomic status. We focus on the adult population, as age is an unpredictable complicator of seemingly simple burn injuries [10,11]. Thus, throughout the burn care pathway, there has been separate development of practices suitable for paediatrics and those fit for adults.

We use LOS as the variable of interest (outcome variable) in this study as it is considered reflective of injury-induced morbidity and the cost and quality of care [12,13]. Furthermore, with a consistent decline in mortality rates [14,15] and, thus, more people surviving very severe burn injuries, there are increased challenges in managing LOS. For this reason, LOS is considered an important performance indicator and outcome measure. To ensure the explainability and reliability of the developed model, we adopt a collaborative decision-making process, involving frequent interaction with medical practitioners.

### 1.1. The Effect of Socioeconomic Factors on Health

The British Medical Association has highlighted that poverty and social inequality adversely affect physical and mental health [16]. Their report goes on to add that these inequalities have implications such as years of life lost, costs to the economy and healthcare challenges. Before the British Medical Association, Marmot and Allen argued that socioeconomic inequalities are key determinants of a patient’s health [17]. These inequalities include material circumstances, the social environment, psychosocial factors, behaviours and biological factors and are influenced by education, occupation, income, gender, ethnicity, and race [18]. A high socioeconomic status leads to accumulated class-related individual resources that translate into higher health literacy (healthier lifestyle) and prevention practices (lower incidence) [19]. Low socioeconomic status has been documented as a risk factor for injury-related hospitalisation, severity and injury-related mortality [20].

In burn care, there is established evidence of the effect of socioeconomic status on the incidence of burn cases [2,21]. This effect is shown in the higher burn incidence in developing countries compared to developed countries [22,23]. In England and Wales, this is also the case: Diguiseppi and colleagues investigated residential fires in inner London and found a higher incidence rate in the most deprived areas [24]. The first exploration of burn injury spanning England and Wales over 2003–2011 saw a potential form of health inequalities due to socioeconomic factors reflected by geographical location [25].

Some researchers have found an association between a patient’s socioeconomic characteristics and LOS for general admission and other specific diagnoses [26,27,28]. Even though socioeconomic status has been shown to influence the incidence rate of burn injuries, there is only little (although growing) evidence of the influence of income, race, educational attainment, housing status and other socioeconomic factors on the outcome of a burn injury [29,30,31,32]. This slow growth of evidence has been due to a lack of data, which includes variables capturing a patient’s socioeconomic status.

The index of multiple deprivation (IMD) is a composite measure that combines different domains of deprivation to produce an overall relative measure of deprivation [33]. The deprivation domains are income, employment, health deprivation, education, barriers to housing, crime and living environment. As a relative measure of deprivation, it can tell us if one area is more deprived than another but not by how much. Additionally, they are not measured on an individual level but at the level of the individual’s social environment, which may limit its ability to capture fine-grained, individual-level detail on socioeconomic status. Hussain and Dunn investigated burn mortality rates and looked at the effect of socioeconomic status using IMD as a proxy [31]. They found a significant increase in the risk of death with every quintile reduction in IMD score, where lower quintile scores indicate higher deprivation.

Understanding the effect of socioeconomic status on LOS would unveil the relationship between a patient’s living environment and the speed of burn recovery. Katanien highlights that class differences in health also imply differences in lifestyle [34]. Thus, understanding the relationship between socioeconomic status and burn recovery may shed light onto the patients’ desire to get back to their living environment. Alternatively, socioeconomic status could also determine the type of burn injury. Injury type is known as a critical determinant of burn severity, which, in turn, influences LOS. A better understanding of these possible influences of socioeconomic factors on burn recovery would ultimately aid in an improved care pathway, leading to better patient outcomes.

### 1.2. How Adverse Mental Health Could Impede Recovery

Mental health can be considered a possible marker for resilience, which motivates our interest in understanding the association of a burn patient’s mental health on their LOS. Anecdotally, “motivated” patients are believed to recover faster and make more gains than patients that are less enthusiastic for treatment. A study that systematically reviews literature found evidence of the adverse effect of stress in impeding wound healing [35]. The review included studies on acute and chronic clinical wounds, experimentally created punch biopsy and blister wounds, and minor damage to the skin caused by tape stripping.

In burn care, there is growing evidence of the adverse effect of mental health factors on burn injury [36,37,38,39,40], both in terms of incidence and recovery. Patients with pre-existing psychiatric illness may suffer more severe burn injuries, which occur due to several reasons, including confusion, carelessness, alcohol or drug intoxication, deliberate self-harm and attempted suicide [38]. The mechanisms impeding burn recovery among mentally impaired patients may range from decreased hope or motivation to participate in one’s recovery to maladaptive behaviours, including aggressive acts and agitated movements that may disrupt treatment [41]. Crucially, it was identified that stress impedes the initial inflammatory phase of wound healing and increases susceptibility to wound infection by the impairment of cellular and humoral immunity [37,41]. Furthermore, the mental state of patients may impact their lifestyles, such as poor diet, smoking and physical fitness, all of which have an impact on recovery [38].

### 1.3. The Interaction of Mental and Socioeconomic Status

A patient’s willingness to return to their living environment—reflective of their socioeconomic status—and to participate in their recovery—reflective of their mental health—reveals the potential interrelationship between their mental health and socioeconomic status. As summarised by a meta-analysis of 56 research papers, Lorant and colleagues found evidence of an association of high psychiatric morbidity and low socioeconomic status [42]. Those found in the lower SES groups have a higher risk of poor coping styles, stress exposure and weaker social support [43]. According to the Marmot review, there is evidence of a relationship between a patient’s socioeconomic status and their psychosocial and behavioural state, which affects lifestyle, social integration and self-esteem, all of which are detrimental to recovery time [17]. This evidence highlights the potential interrelationship of a patient’s socioeconomic status to their mental health and the influence both may have on a patient’s LOS.

**Figure 1:**
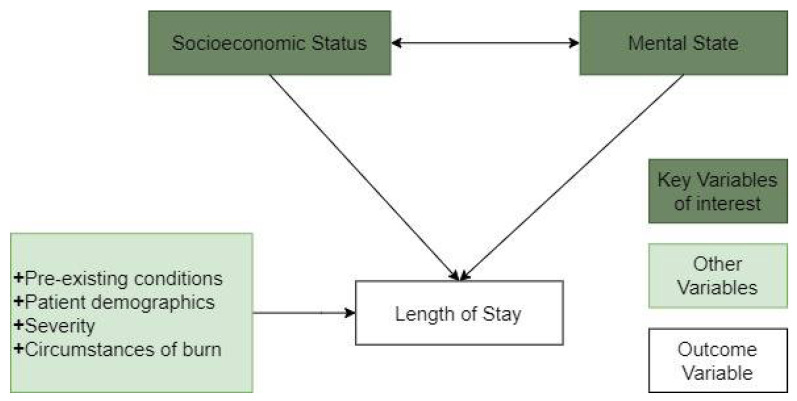
Relationship between socioeconomic status, psychological state, and other factors on patient length of stay (LOS).

To investigate the research questions raised in Section 1.1, Section 1.2 and Section 1.3, we make three hypotheses, as depicted in Figure 1 and summarised below.

**Hypothesis** **1.** *Segmentation by socioeconomic status or mental health state produces groups that are more homogenous with respect to LOS than the nonsegmented population.*

**Hypothesis** **2.** *We suspect an interrelationship between a patient’s socioeconomic status and their mental health and that both interactions have an impact on LOS.*

**Hypothesis** **3.** *If Hypothesis 1 is supported, we expect that the relative influence of severity on burn patients reduces as the presence of adverse mental or socioeconomic state increases.*

In evaluating the hypotheses, this study aims to understand, in the context of burn patients, the association of mental and socioeconomic status on length of hospital stay. We also study if there exists an interrelationship between mental health and socioeconomic status. We conduct this study by adopting a collaborative decision-making process involving the Manchester Centre for Plastic Surgery and Burns, part of the University Hospital of South Manchester. We refer to the collaborators, going forward, as burn care specialists.

## 2. Materials and Methods

### 2.1. Data

This study uses anonymised patient-level data from all burn units in England. The data covers the period 2003–2019, comprising just over 50,000 admitted patients, and was extracted from the international Burn Injury Database (iBID) [44]. Patient information was collected by clinicians and nurses from the first contact with a burn service through to rehabilitation and any late reconstruction procedure [25]. The dataset includes all types of burn injury patients who attended a burn service. The dataset excludes burn injury patients who died before reaching a burn service and those with minor injuries that were cared for in the community and in hospitals that do not have specialist burn services.

We focus on the adult population (age ≥ 16) as pediatric patients are more likely to be subjected to unpredictable complications, even with seemingly simple burn injuries, as highlighted by the 2001 National Burn Care Review Report [10]. The report argues and mandates the need for separate burn units for children and adults due to the peculiar needs of children, such as play specialists, teachers, family counsellors and intensive psychosocial support. Thus, the burn care pathway is designed to treat paediatrics separately from adults as young age is a significant complicator. In our previous study on the segmentation of burn patients, we found further evidence that adult and pediatric burn patients are characterised by different variables (such as the source of injury and presence of existing disorders) and have different resource requirements [11]. Consequently, to allow for more robust predictions, in this study, we discard all cases of age less than sixteen. The cases removed also included nonsurvival cases as we expect them to have shorter LOS than counterparts who survive. Accurate referral of patients to burn units implies that there was an increased number of admitted minor injury cases that were previously treated in other care settings. Therefore, given our focus on predicting LOS on admission, those with no overnight stay were excluded.

### 2.2. Variables

As stated above, this study considers LOS as the key indicator of outcome due to its ability to reflect injury-associated morbidity, cost, and efficiency. We calculate LOS as the number of days a patient is admitted for (days from admission to discharge). LOS was log-transformed to remove skew. The iBID database [42] gives us access to about 300 variables.

Our goal is to understand what variables drive the prediction of LOS at the point of admission as this will allow for improved patient triaging, resource planning, and smoother patient discharge. We exclude patient information obtained after admission, such as complications and operation duration. Compared to a model that captures the full pathway of a patient, this exclusion will likely lower prediction accuracy, but our focus here is prediction and prediction performance at the point of admission. Other variables were removed with guidance from a burn care specialist; this included variables with a high number of missing values, administrative variables and variables that give information obtained from other variables. Further variable selection was made using LASSO (least absolute shrinkage and selection operator), as described in Section 2.3.

Through collaborative decision-making with a burn care specialist, the variables were also grouped into similar types. In total, for the 107 significant variables, we identified six variable groups, as summarised in Table 1. Four of these variable groups cover pre-existing conditions, demographic factors, and the severity and circumstances of the burn, respectively. These injury attributes provide important insight regarding the source of the burn, the type of burn injury sustained, the activity engaged in when the burn occurred, locality, living circumstances and space where the injury occurred, time from injury to admission and if first aid was administered. The circumstance of the burn also includes an indicator of injury occurrence (for example, through assault or work-related), named injury category. Finally, the two main variable groups of interest in this study are those that relate to socioeconomic status and mental health. IMD serves as a proxy for socioeconomic status, while a group of seventeen variables serve as proxies for mental status. More information on these variables is provided below.

Socioeconomic Status: Socioeconomic status must be appropriately operationalised to allow the analysis of its impact. There are two approaches to studying the influence of socioeconomic status on health disparity: the compositional approach, which measures socioeconomic status by referring to characteristics of the individual, and the contextual approach, which measures socioeconomic status as the characteristics of an individual’s environment [45].

In this research, we have access to a contextual measure of socioeconomic status, which is the IMD index. This index combines variables of income, employment, health, education, crime, housing, access to services and living environment. As noted by Kaplan, it is paramount to study the social environment to understand the day-to-day experience of individuals and groups and how that translates into better or worse health among richer and poorer populations [46]. This is also reflected by the World Health Organization report on health inequalities, which discusses the potential influence of the environment in which people grow, live, and work, age and the systems put in place to deal with illness on health inequalities [47]. The IMD index captures the above aspects, with deprivation defined as the lack of access to opportunities and resources [48]. The iBID database holds the 2004 IMD score, which is at a local authority district level [33]. The score ranges from 0 to 100, with 100 indicating extreme deprivation. The IMD index is published by the Office of National Statistics every 3–4 years; however, the version available to us via the iBID database is the 2004 version.

Mental State: Disorders of the mental state may be caused by changes in biological factors, such as changes of the brain, aberrations of the immune system, and changes in psychosocial factors such as effects of interpersonal relations and negative body image [49]. These disorders affect a person’s thinking, feelings and behaviours.

In this study, we use some of the major indicators of an individual’s mental health, as highlighted by the WHO ICD-10 Classification of Mental and Behavioral Disorders Addiction [50]. The relevant variables used in this paper indicate the presence/absence of certain mental disorders before the burn injury, i.e., pre-existing disorders, and others captured by identifying categories of burn linked to adverse mental health. We considered 15 pre-existing mental health variables: alcohol abuse, substance abuse, psychiatric disorder, depression, post-traumatic stress disorder (PTSD), anxiety, personality disorder, mania, schizophrenia, self-harm, attention deficit hyperactivity disorder (ADHD), eating disorder, dementia and learning difficulty. The pre-existing mental conditions are captured in the iBID database through patient indication and ascertained using access to available health records. Two other adverse mental state variables can be identified from the category of burn occurrence, namely, whether the burn was caused by self-infliction or a suicide attempt.

### 2.3. Analytical Pipeline

To ensure the developed model is explainable and reliable, while predicting LOS accurately, we developed an analytical pipeline that entails a collaborative and iterative decision-making process. The analytical pipeline includes data preprocessing, segmenting patients by their socioeconomic and mental status, and the building and testing of least absolute shrinkage and selection operator (LASSO) regression models. To ensure result validity and reliability, the analytical pipeline includes *k*-fold cross-validation. This involves the training and evaluation of the model across *k* random splits of data into test (10%) and training (90%) datasets to understand the variability of evaluation metrics and coefficients of significant predictors across these different “folds”. The test dataset is a hold-out test dataset used to test the reliability of results, i.e., the predicted LOS is compared to the expected LOS to identify the model’s ability to predict the LOS for newly admitted patients accurately.

**Figure 2:**
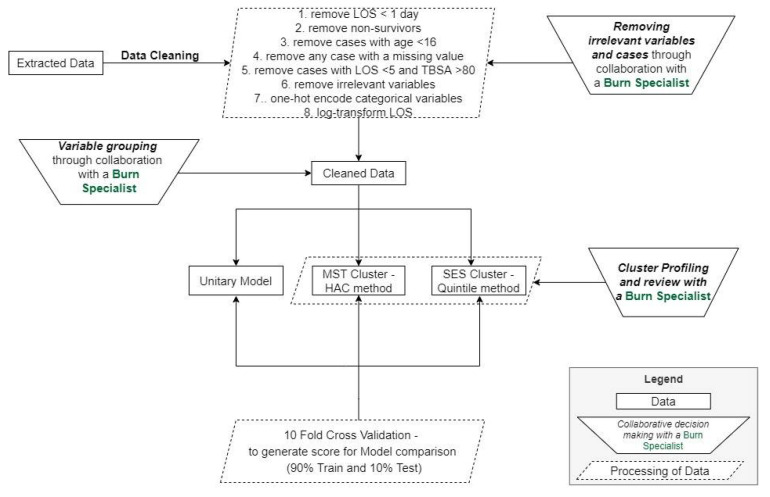
Analytical pipeline showing data cleaning/preprocessing to model deployment and testing.

As indicated in Figure 2, the first step is data cleaning and preprocessing, which includes removing cases and variables that are not relevant, as detailed in the Data and Variables Section. With insight from a burn care specialist, cases with TBSA over 80 percent but an LOS under five days are flagged as outliers, as it is expected that such cases should have a higher LOS. In summary, the sample size was reduced from over 50,000 to 19,837 patients after applying the identified exclusion criteria (removal of patients with age less than 16, patients with no overnight stay, nonsurvivor cases, outlier cases, and cases with missing data). The data preprocessing step also contains one-hot encoding, where categorical variables are converted into binary variables, excluding the first category in each variable to avoid the dummy variable trap. As a result of one-hot encoding, the number of variables available for model building increased from 107 variables to 308.

To explore our hypothesis of increased homogeneity in terms of LOS, two segmentations were created and the predictive models were developed separately for the identified segments. The first segmentation uses the socioeconomic status variable (SES-based clusters), and the second segmentation uses all seventeen variables that reflect a patient’s mental health (MST-based clusters). For comparison, a predictive model with all adult patients (no segmentation) was created (unitary model).

Due to the availability of a single segmentation variable, the quintile method segmentation of data into five equal parts was adopted for the SES-based cluster set. This method allows for segments that are explainable. It is consistent with approaches found in the literature and used by government bodies, such as the Office for National Statistics and Public Health England, where IMD is typically grouped by dividing the sample into five parts: 0–20, 20–40, 40–60, 60–80 and 80–100 [31,51,52]. However, as IMD is a standardised measure that reflects the relative level of deprivation of an area, the parts are only equal when segmenting population data. In line with this, we segment cases into a group of five; the most deprived group is IMD score ≥ 80, and the least deprived group is IMD score < 20. Given that the burn care data does not reflect the general population, we would have unequal group membership across segments.

We identified the MST-based clusters using all variables that reflect mental health. Group membership was determined using a clustering algorithm as there was no prior knowledge regarding an appropriate cluster number nor an established membership criterion for creating homogenous groups in terms of mental health. Clustering is an unsupervised learning technique for partitioning sets of data items into several disjoint clusters based on a similarity measure [53,54]. In particular, this paper uses the average link hierarchical agglomerative clustering (HAC) method [53], a well-established technique in unsupervised machine learning, previously used in our research on segmentation of burn patients [11]. The average-link linkage criterion was chosen as it is appropriate for noisy data and reduces the probability of uneven clusters. In average link, homogenous clusters are obtained by calculating the distance between clusters as the average of pairwise distances between all observations in the two clusters.

One of the advantages of HAC for our application is its ability to work with any distance metric to identify the similarity between data points [55]. In our case, the distance between cases (patients) is measured using the Gower metric, which was developed for datasets containing a mix of categorical, binary and numerical variables [56]. For each variable type, it uses the most appropriate distance metric. For MST-based clustering, given that the variables are all binary, the Dice coefficient is used to calculate the distances.

Given the absence of prior knowledge of the number of clusters in the dataset, model selection was supported using internal validation variables and the interaction with burn care specialists. First, we used the Silhouette and Calinski–Harabasz scores to determine the most promising number of clusters. The Silhouette score of a data instance measures how similar an object is to elements in its cluster compared to elements in the nearest cluster [57]. The Calinski–Harabasz score, also known as the variance ratio criterion, is the ratio between within-cluster dispersion and between-cluster dispersion [58]. The partition identified at this stage was passed on for further validation to a burn care specialist to ensure that segments were explainable and clinically relevant.

Due to the potential presence of multicollinearity in the data, for example, the presence of numerous variables that represent severity, LASSO [57] was selected to build the models. LASSO is a type of linear regression, differentiated by its regularisation characteristics [59]. In particular, we use the least angle regression (LARS) LASSO [60]. LARS LASSO is an efficient stepwise variable selection algorithm. As a regularisation algorithm, it uses a tuning parameter to shrink the coefficients of the nonsignificant variables to zero, thus removing nonsignificant variables from the model. Where there are highly correlated variables, it tends to choose one variable only [61]. Therefore, the method is more likely to prevent overfitting, which is another motivation for using LARS LASSO. For our LARS LASSO model, to ensure low bias and variance in the model, 20-fold cross-validation was used to ascertain the best value of the tuning parameter, lambda, for the regularisation process.

The LARS LASSO algorithm was fitted on the two cluster sets and the unitary model.

SES-based clusters: the socioeconomic variable used for clustering was excluded as an independent variable in the LASSO model; thus, all SES-based models included 307 independent variables

MST-based clusters: In this cluster set, all mental health variables were excluded; thus, the MST-based models were fitted using 291 independent variables.

Unitary model: The LASSO model fitted for the unitary model contained all 308 independent variables, plus three interaction terms. The interaction terms were included to enable the evaluation of how the presence of key aspects (mental health, socioeconomic status and burn severity) is associated with LOS. The variables chosen to represent each of those aspects were selected by drawing on the literature. For socioeconomic status, IMD was used, while TBSA was used for severity. To accurately represent mental status, a new variable, *MST_count*, which represents the count of adverse mental health, including pre-existing mental disorder and the other two mental health variables identified from the category of burn, was created. The three interaction terms are TBSA*MD, TBSA*MST_count and IMD*MST_count.

Given the adoption of 10-fold cross-validation models, the generated coefficients from each model were averaged to identify the expected population coefficient. Subsequently, the effect size (which expresses variable importance) was obtained for each variable by standardising the coefficients from the LASSO model. The coefficients from multiple linear models represent the relationship between the given variable and the target variable, assuming all other variables are constant. With the inclusion of variables with different units, there is a need to standardise the generated coefficients. This is done by multiplying each variable’s coefficient by the standard deviation of the variable. This means that the greater the variance of a variable, the larger the standardised coefficient (and therefore the impact of the variable on the output), all else being equal.

Model fit and performance were ascertained using *R*-squared (*R*^2^) and weighted root mean square error (*wRMSE*) on the training and test data. To achieve objective comparison across segmentations, an adapted root mean square error measure was devised. We calculated the weighted sum of RMSE across all clusters, with weights reflecting the proportion of the data covered by each cluster (Equation (1)):

(1) $$wRMSE=\overset{K}{\underset{k\;=\;0}{\sum }}\frac{n_{k}}{n}\;\sqrt{\;\frac{1}{n_{k}}\;\overset{n_{k}}{\underset{i\;=\;1}{\sum }}{\left(Y_{i}-{\hat{Y}}_{i}\right)}^{2}}$$

Here, $n_{k}$ is the sample size in cluster *k*, n is the total sample size of all clusters in a cluster set, ${\hat{Y}}_{i}$ is the predicted value and $Y_{i}$ the observed value.

To understand the proportion of the variance in the dependent variable that is predictable from the independent variable, we measure $R^{2}$ in Equation (2):

(2) $$R^{2}=1-\left[\frac{\sum_{i\;=\;1}^{n}{\left(Y_{i}-{\hat{Y}}_{i}\right)}^{2}}{\sum_{i\;=\;1}^{n}{\left(Y_{i}-{\bar{Y}}_{i}\right)}^{2}}\right]$$

where ${\bar{Y}}_{i}$ is the mean of observed values in the training data.

## 3. Results

**Figure 3:**
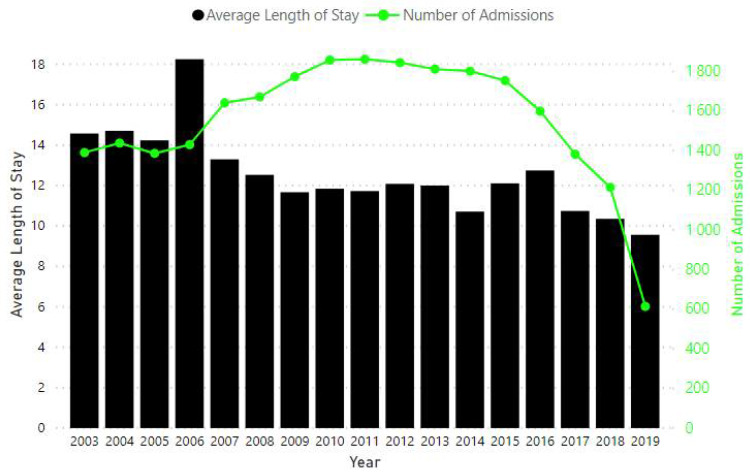
Number of admissions and change in the average length of stay from 2003 to 2019.

In total, over 19,000 admitted adult patients with at least an overnight stay were analysed. There is a consistent decline in average LOS over time (Figure 3). A reduction in the number of admitted patients can be seen from 2011.

Our initial data exploration provides some first insight into the variability in LOS and the key drivers behind it. The LOS of adult patients ranged between a day and 455 days, with a median of five days and an interquartile range (IQR) of eleven days. This high variability highlights the potential benefits of constructing patient segments. We find that there were more males admitted (68%) than females (32%), with the median LOS for women at seven days being higher than that of males at five days. The age of admitted adult cases ranged from 16 to 113 years old, and LOS varied with age: the older the patients, the higher the median LOS.

**Figure 4:**
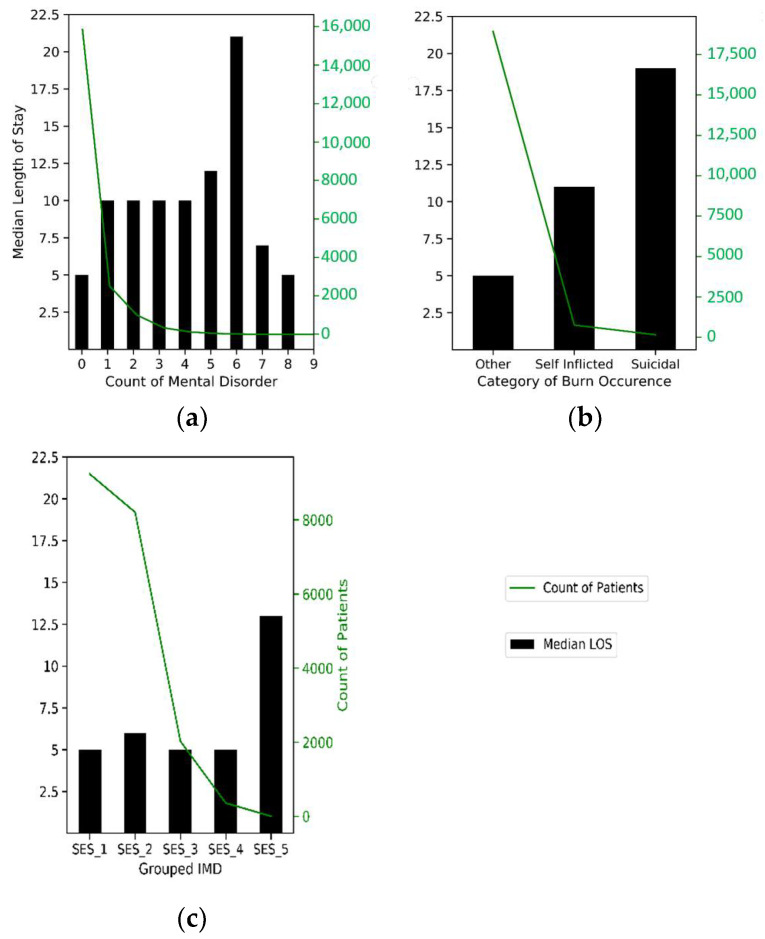
Patient profile by key variables of interest. (**a**) This shows varying median LOS by the number of pre-existing mental disorders. (**b**) This shows varying median LOS by category of burn grouped into two mental states and one other state (nonmental-state-related). (**c**) With deprivation increasing from left to right, this shows varying median LOS by socioeconomic status (SES).

Of the adult patients, 20 percent had one or more pre-existing mental condition. Patients with a mental-health-related pre-existing condition have a median LOS of 10 days—the lowest median was found for patients with PTSD, at five days, and the highest was amongst those with dementia, at 17 days. The higher the number of pre-existing conditions, the higher the median LOS except for those with 7 to 9 disorders with a median LOS of seven days and five days, respectively. Patients with 1 to 4 pre-existing mental health disorders have a median LOS of 10 days, while those with no pre-existing mental health conditions have a median LOS of 5 days (see Figure 4a).

Exploring how the burn injury was inflicted, 95 percent of cases were caused accidentally or through assault and had a median LOS of five days (Figure 4b). Patients with injury infliction from a suicide attempt accounted for 1 percent and had a median LOS of 20 days, while those due to self-infliction accounted for 4 percent, with a median LOS of 11 days. Exploring IMD, where 0 IMD indicates relatively no deprivation and 100 indicates extreme deprivation, there is a slight increase in LOS as the IMD score increases and a significant correlation with LOS. From Figure 4c, median LOS is the same at five days for the first, third and fourth SES quintile groups and sixth in the second group (20–39 IMD score). As expected, the median LOS is at the highest for the most deprived group (SES_5), with a value of 13 days.

These striking differences suggest support for our hypothesis that segmentation by socioeconomic status or mental health produces groups that are more homogenous with respect to LOS than the nonsegmented population. We now proceed to consider whether a segmentation by socioeconomic status and another by the mental health of patients can reduce LOS variability and result in better prediction compared to the unitary model. For the SES-based cluster set, group membership was identified by quintile classification of IMD. Given the IMD range of 0–100, this creates five groups, the first being the least deprived group (IMD < 20); the second group has IMD of 20 to 39, the third is 40 to 59, fourth is 60 to 79 and the fifth group has the most deprived cases, with IMD ≥ 80. Though it is expected, when analysing the population in England, that these quintile groups should have equal membership, due to low admission into burn care units from the two most-deprived groups, we see low case membership in SES_4 and SES_5. Thus, to avoid creating a heterogeneous group in terms of deprivation indicators, cases assigned to these groups were removed from the analysis altogether. This further reduces the sample size from 19,837 patients to 19,474 patients.

**Figure 5:**
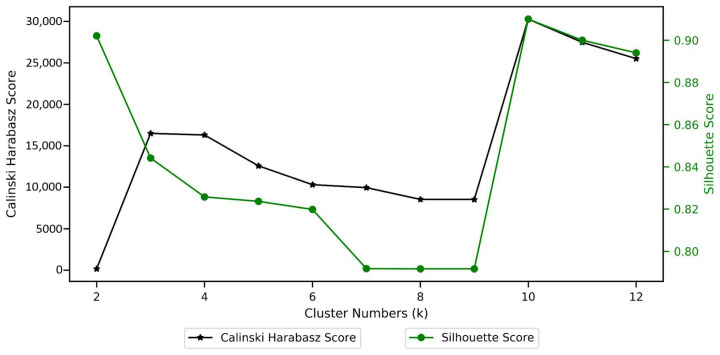
*k*-Selection using Calinski–Harabasz and Silhouette scores.

As discussed in the Analytical Pipeline section, the MST-based cluster set was identified by adopting an approach that allows for identification of an optimal number of groups and cluster sets in terms of the seventeen mental state variables. The most suitable number of groups has been identified from examining Figure 5, which shows that at *k* = 10, group separation on mental state variables is at its maximum.

Cluster characterisation further showcases a clear separation and easy-to-describe grouping. The first group comprises all cases with no mental-health-based disorder, and the second group comprises those with one mental-health-based disorder, which is either a pre-existing disorder or derived from the category of injury infliction. The third group comprises those patients with two mental health disorders—either as two pre-existing mental conditions or as one pre-existing mental condition and injury infliction by a suicide attempt or self-infliction. The remaining seven groups comprise patients having three or more mental health disorders, with each group distinguished by the increasing number of mental health conditions present. However, these groups are small, which means that LASSO modelling is not feasible as the number of variables exceeds the group membership.

A burn care specialist was, therefore, consulted to review the group membership. A collaborative decision-making process led to the adoption of a less granular two-group structure, representing the presence or absence of mental state disorder, respectively. This segmentation was judged to be easy to interpret and clinically relevant by the burn care specialist. Thus, for this paper, the small clusters (2–10) were merged to produce a single cluster of adverse mental state.

In summary, the segmentation steps produced *k* = 10 (with *k* ≥ 2 classes merged) for MST-based segmentation and *k* = 5 (with the fourth and fifth cluster excluded from LASSO model-building due to low membership) for SES-based clustering. The final groups and their characteristics are shown in Table 2. With regards to LOS and TBSA, the two MST-based clusters are different as its mean and standard deviation reflect different LOS, TBSA and age. We see an increased average LOS as the number of adverse mental states increased. This clear distinction across clusters is not found in SES-based clusters, most likely due to IMD not being an individual-level measure of deprivation. Having identified the MST-based and SES-based patient groups, these and the unitary adult group were used to build our LASSO models. The interpretation of the model coefficients will reveal if the impact of severity reduces with an increased presence of adverse socioeconomic/mental status, depicted by decreased severity effect size. Similarly, the significance of the interaction terms will provide insight regarding the presence of an interrelationship between severity, mental state, and socioeconomic status.

**Figure 6:**
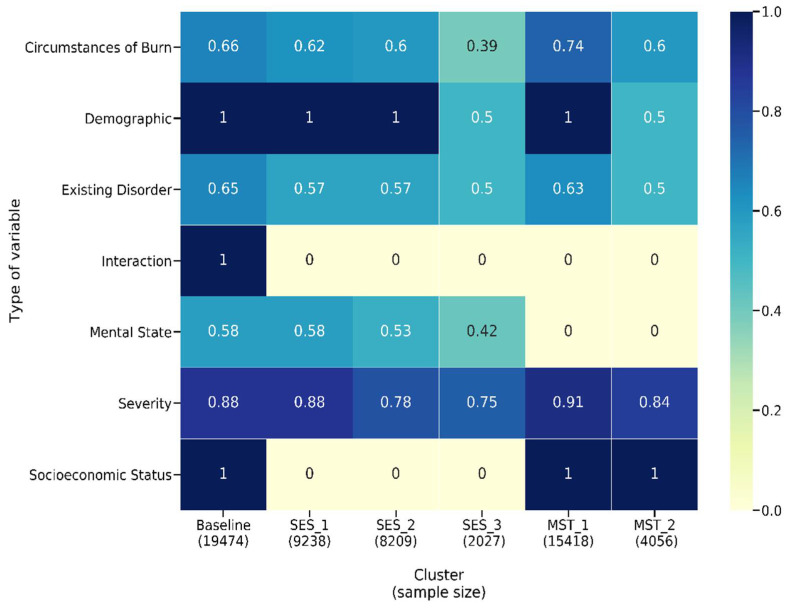
Heatmap showing the proportion of variables that have a nonzero coefficient in each type for MST-based, SES-based and unitary models.

**Figure 7:**
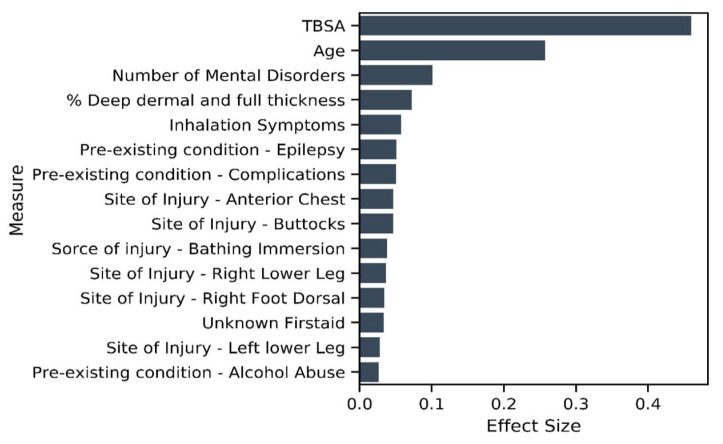
Variables of importance—top 15 contributors from 212 significant variables in the unitary model.

The results of the unitary model indicate 212 significant variables. Evaluating the summed effect of the severity variable group, as expected, it had the highest sum at 0.92 compared to all other variable groups. The majority of variables describing severity (except for indicators of burn occurrence in the face or to the right forearm) had a positive effect size, indicating higher severity is associated with a longer LOS. In total, 88 percent (Figure 6) of severity variables have a nonzero coefficient. We found that eight of the top fifteen variables that predict longer LOS in the unitary model are severity variables (see Figure 7). As might be expected, TBSA is the most important predictor of burn. We found 65 percent of the existing disorder variable type as significant and two variables included in the top 15 contributors to longer LOS. As expected, increased age leads to an elevated risk of increased LOS and is the second most important predictor of LOS.

Focusing on the aspects of interest in this paper, the results show that IMD (and, therefore, 100 percent of variables related to socioeconomic status) and 58 percent of the mental state variables (depression, personality disorder, schizophrenia, PTSD, substance abuse, psychiatric disorder, anxiety, dementia, learning difficulty, alcohol abuse and a count of pre-existing mental disorder) are determined to be significant predictors of LOS. IMD’s effect size is 0.02. In comparison, mental state variables had a summed effect of 0.14. Many of the mental health variables have a positive effect size, and two of them are amongst the top 15 predictors of longer LOS. In the unitary model, all variables capturing a patient’s demographic (age and gender), are also significant predictors of LOS. The three interaction terms, MST_count*TBSA, MST_count*IMD and IMD*TBSA, are all significant predictors, providing evidence for our second hypothesis as the significant interactions show evidence that the effect of a patient’s mental health state on LOS is different for different values of socioeconomic status and vice versa. Similarly, the effect of TBSA on LOS varies with varying values of socioeconomic status and mental health state. The MST_count*TBSA coefficient reveals a negative relationship with LOS, which reveals that the effect of TBSA (severity) on LOS will decrease with the increased presence of mental health disorders.

**Figure 8:**
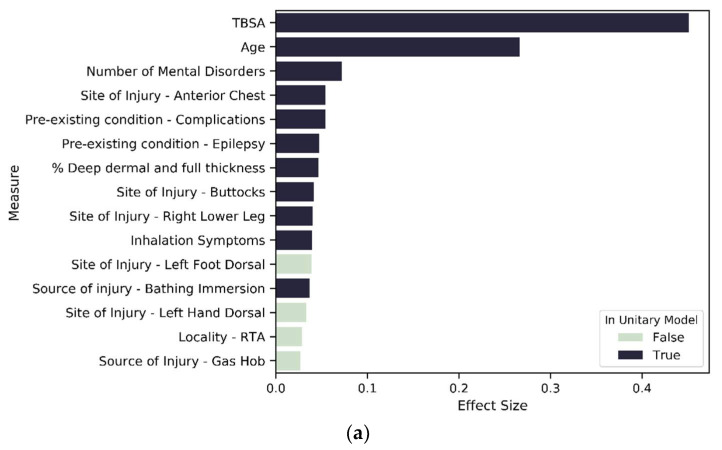

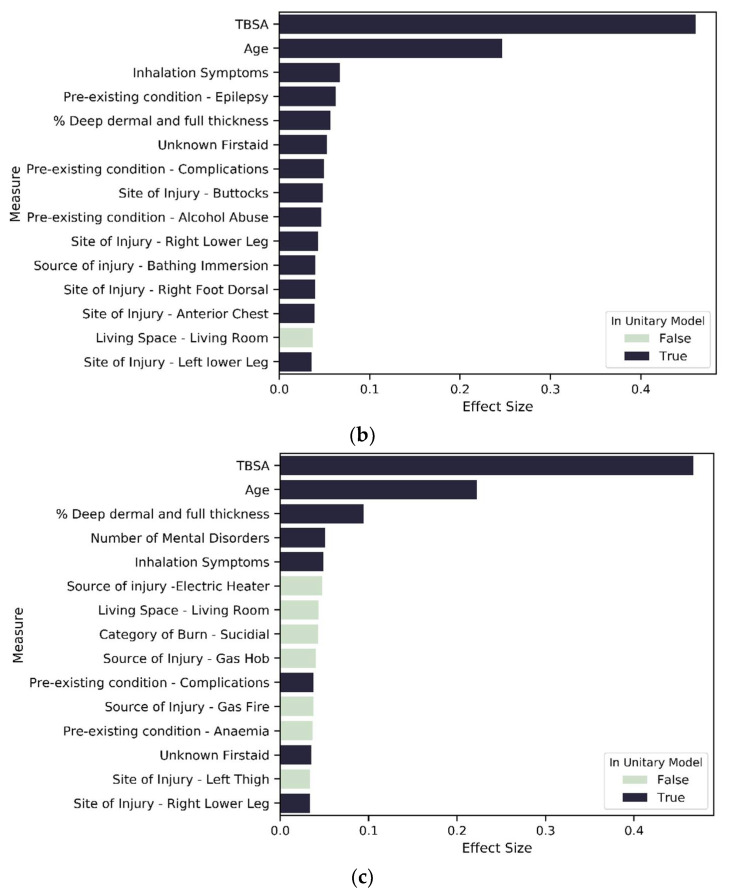
Variables of importance: (**a**) top 15 contributors from 196 significant variables in SES1; (**b**) top 15 contributors from 188 significant variables in SES2; (**c**) top 15 contributors from 137 significant variables in SES3.

**Figure 9:**
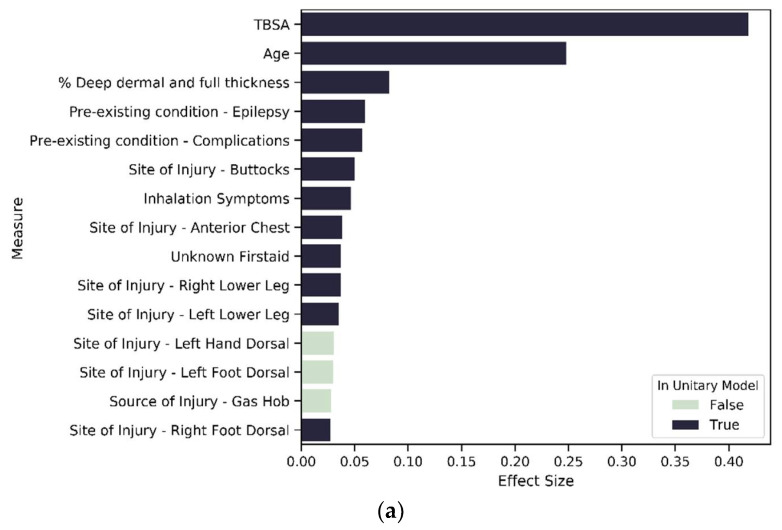

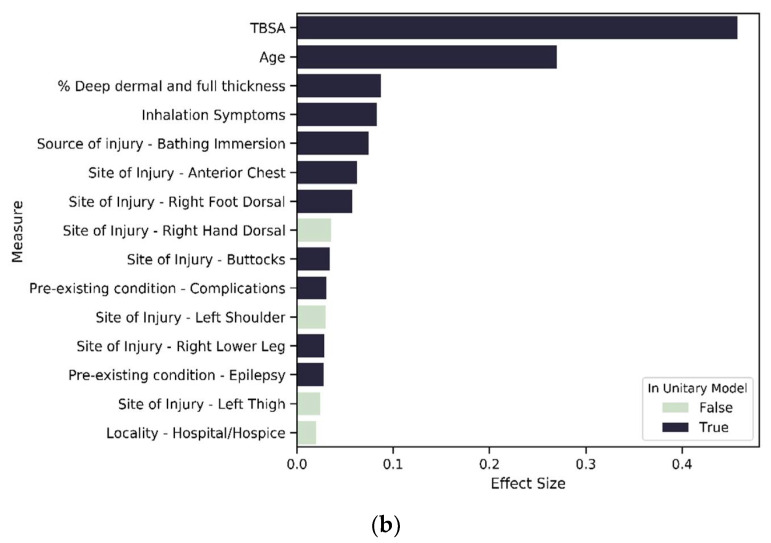
Variables of importance: (**a**) top 15 contributors from 215 significant variables in MST1; (**b**) top 15 contributors from 177 significant variables in MST2.

To investigate our third hypothesis, we compared the cluster-specific models, exploring the proportion of variable types that are significant predictors of LOS. Across all models—unitary (Figure 7) and cluster wise (Figure 8 and Figure 9)—TBSA and age are the two most important predictors of LOS. Inhalation symptoms, percentage of full dermal burn and pre-existing complications also appeared as a top-15 predictor in all the models, although in varying order. All clusters, including the unitary model, have a proportion of severity, pre-existing disorders, demographic factors and circumstances of burn as a significant predictor.

For SES-based clusters, the proportion of significant predictors across the variable types varies across the cluster set, indicating that deprivation quintiles differ in terms of the impact of specific variables on LOS. More specifically, the proportion of variables that are significant in each variable type reduces as deprivation increases. Most notably is severity, mental state and existing disorder. For the most deprived group—SES_3—seven of the top 15 variables of importance are not found in the unitary model (Figure 7 and Figure 8c). These seven variables are related to living circumstances and the source of injury. SES_2 has all variables found in the unitary model included within the top 15 predictors, except for the variables that indicate the living space in which the burn occurred (Figure 7 and Figure 8b). SES_1 was mostly similar to the unitary model; the difference in both models is that SES_1 has a variable indicating living circumstances as significant (Figure 7 and Figure 8a).

In the MST-based clusters, we observe a reduction in the proportion of significant severity variables as the extent of mental disorders increases (91 percent for no mental disorder and 84 percent for mental disorder group—see Figure 6). This pattern is found across all variable types except for IMD, which is a significant predictor in both MST models but has a higher effect size in MST_1 compared to MST_2. For MST_1 and MST_2, the 10 most important predictors are predominantly variables that reflect severity, age inhalation symptoms and previous complications, except for MST_2, where locality of burn is the tenth most important predictor (Figure 9a,b).

**Figure 10:**
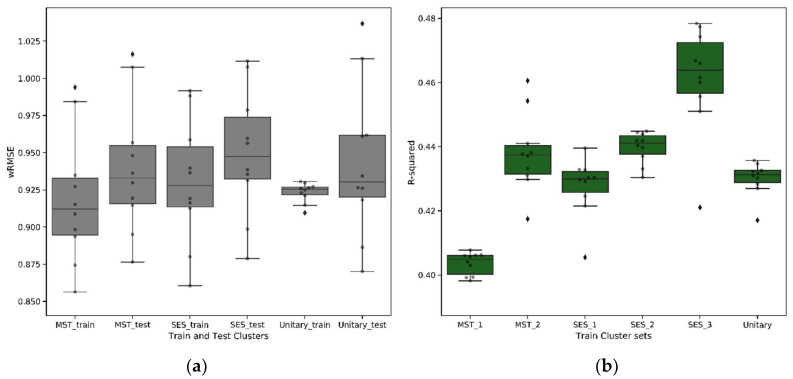
Evaluation metric: (**a**) weighted root mean square error (wRMSE) from each k-fold model (train and test), averaged across a cluster; (**b**) R-squared on train models on each cluster set.

Comparing the models using wRMSE, we found no significant difference in the performance of the cluster-wise models compared to the unitary model (Figure 10a). This is an important finding as it suggests that the cluster-wise models have captured the most important influence of mental health and socioeconomic status. With mental health and socioeconomic status being significant in the unitary model, prediction accuracy of the cluster-wise models were not impacted when these key variables were removed. The cluster-wise models bring clear benefits in terms of the complexity and explainability of the resulting regression models and the potential to exploit the underlying segmentation in a personalised care pathway and the creation of reimbursement models. Except for MST_1, the unitary model has the highest number of significant variables, whereas the cluster-wise models give rise to simpler models, highlighting the improved homogeneity of these patient groups. This is also evident from Figure 10b, which illustrates that, except for MST_1, the cluster-specific regression models have a higher $R^{2}$ score than the unitary model, indicating a higher ability to explain the variability in LOS through a regression function.

## 4. Discussion

In this study, IMD and a set of mental health variables (17 in total) were examined to ascertain the association between these factors and LOS. These variables include pre-existing mental health conditions in patients and cause of injury due to self-infliction or suicide. IMD was used as a measure of socioeconomic status, as done in previous burn care research (see, e.g., [31,62,63,64]), capturing contextual variables of deprivation. The results of our unitary model support previous burn care research, highlighting that TBSA, age, inhalation injury and depth of burn are the most important predictors of burn [65,66,67,68,69]. We further provide evidence for the association of mental state variables with LOS, showing that the number of mental state conditions is a significant, top-three predictor of LOS.

For mental state, its influence on LOS was observable in the exploratory analysis, as depicted in Figure 4, in the form of a trend of increased median LOS with an increased presence of adverse mental health in a patient. Median LOS for injury caused by a suicide attempt or self-infliction was found to be higher for these patients compared to those caused by accident or assault. This exploratory analysis reveals that patients with adverse mental health are more likely to spend a long time admitted in hospital than patients without these conditions. This finding is in line with anecdotal evidence and with previous research on the impact of adverse mental/psychological state on the LOS of burn patients [37,38,39,41,70]. Our research builds on this previous work in two ways. Firstly, we used a larger sample size compared to previous research. Secondly, with the adoption of cluster-wise models, we explored how the impact of variables on LOS changes with different levels of adverse mental health status.

The MST-based cluster result gives better insight into the relationship between LOS and mental disorder and its interrelationship with socioeconomic status. A clear trend is shown: with an increasing presence of mental disorder, there is a reduced number of burn severity variables that significantly predict LOS. The cumulative effect size reveals a higher association of LOS and severity variables in the no-mental-disorder group compared to the groups with any form of mental disorder. This begins to answer Hypotheses 1 and 3, indicating greater homogeneity in groups of patients with similar mental states compared to the general adult burn patient population. The resultant higher cumulative effect size of severity variable in the no-mental-disorder group compared to those groups with any level of mental disorder further evidence the need for grouping to obtain better homogeneity. Therefore, severity, as well as mental disorders, need to be triaged effectively to enable better injury management and outcomes. Our results corroborate the work of Tarrier and colleagues, who found that patients with pre-existing mental conditions and self-inflicted injuries are associated with difficult clinical management and higher LOS [38]. The analysis of the cluster profile (Table 2) revealed an association of increased mental disorder to increased TBSA; this is further evidenced by the significance of the interaction term that accounts for these two variable types, thus revealing a potential interrelation with these two variables and the need for a personalised triaging process which takes into account a patient’s mental state.

For socioeconomic status, our research goes further than previous research that evaluated and found evidence of the influence of socioeconomic status on the incidence of burn [21,62,63]. Rather than considering the impact on incidence rate, here, we investigate the impact of socioeconomic factors on patient recovery by considering the relationship between IMD and LOS. The SES-cluster-based results show a similar trend. The least deprived group have the highest proportion of severity variables as significant, and the most deprived group has the lowest number of severity variables as significant. We also found that in the SES-based cluster, variables that reflect the mental health of patients were found as significant predictors. This may be linked to a potential interrelationship between mental state and socioeconomic status that was further evidenced in the unitary model where the interaction term of mental state and socioeconomic variables had a significant positive effect, revealing that with an increased presence of adverse mental health conditions, the effect of deprivation increases. This supports the 2010 Marmot review, which found evidence of the influence of socioeconomic status on an individual’s psychosocial and behavioural state [17]. The difference in the top 15 predictors in the most deprived group compared to the unitary model shows the impact of the living circumstances of patients on burn outcome as these have a higher effect in this group, thus revealing the influence of an individual’s living environment on an increased probability of a more severe burn injury. Researchers have found that living environment and other socioeconomic facts increase burn incidence and its severity [29,30,31,32]. Our research corroborates this and, for the first time, identifies certain living circumstances—which indirectly reflect a patient’s socioeconomic status—as top predictors of LOS.

Our study does have some limitations. A key limitation is the use of IMD as a proxy for a patient’s socioeconomic status. As IMD is not an individual-level indicator but instead represents relative deprivation of small areas, an individual-level indicator would have better reflected a patient’s socioeconomic status at the point of admission. With IMD scores updated every 3 to 4 years, ideally, each patient’s score should be recorded at the point of admission. However, we used the 2004 IMD score for all patients. Future research should use individual-level indicators of socioeconomic status at the time of admission to better reflect a patient’s socioeconomic status.

## 5. Conclusions

Our study suggests segmentation of burn patients in terms of their socioeconomic status or mental state. The groups we have identified are easy to explain and facilitate a better understanding of patients by clinicians. In particular, the usefulness of these groups was evaluated with the development and comparison of a set of cluster-specific regression models. Evaluating significant predictors of LOS, we found that with the adverse mental state or socioeconomic status of a patient, the influence of severity (established predictors of LOS) reduces. This finding highlights the importance of factors such as socioeconomic status and mental health on a patient’s recovery.

The adoption of a collaborative decision-making process in our research ensured the production of models that allow easy understanding by clinicians and, thus, the ability to understand the reasoning of model outcomes. With explainable results, this study will aid burn units in better triaging patients by personalising their care pathway, such as taking their mental state and socioeconomic status into account.

## Figures and Tables

**Table 1:** Variables used in this study grouped by type, and an indication count for categorical variables.

Type of Variable (Count)	Variables (Number of Categories in the Categorical Variables)
socio-economic status (1)	IMD.
mental state (17)	self-inflicted injury, injury from suicidal attempt, number of existing psychological order, alcohol abuse, substance abuse, psychiatric disorder, depression, post-traumatic stress disorder (PTSD), anxiety, personality disorder, mania, schizophrenia, self-harm, attention deficit hyperactivity disorder (ADHD), eating disorder, learning difficulty, dementia
pre-existing conditions (47)	non-insulin-dependent diabetes mellitus, insulin-dependent diabetes mellitus, epilepsy, asthma, bronchiolitis, upper respiratory tract infection, urinary tract infection, nonspecific viral illness, chronic obstructive pulmonary disease, emphysema, preadmission bacteria, perivascular disease, ischemic heart disease, past myocardial infarction, hypertension, cardiac failure, congenital heart disease, heart valve disorder, cardiac dysrhythmia, neoplasm, metastatic disease, past cerebrovascular, hemiplegia, past deep vein thrombosis, poor hearing or deafness, poor eyesight or blind, mild hepatic dysfunction, moderate to severe hepatic dysfunction, renal dysfunction, cerebral palsy, paraplegia, spina bifida, motor disability immobility, sensory disability, multiple sclerosis, 1st trimester—pregnancy, 2nd trimester—pregnancy, 3rd trimester—pregnancy, hepatitis B, hepatitis C, human immunodeficiency virus, anemia, clotting disorder, immune suppression, leukemia lymphoma, resus, previous complications
demographic factors (2)	gender (2), age
severity (31)	total burn surface area (TBSA), percent superficial burn, percent deep dermal burn, inhalation symptoms, body area burn occurred (face, neck, scalp, anterior chest, posterior chest, right shoulder, left shoulder, right upper-arm, left upper-arm, right forearm, left forearm, right-hand dorsal, right-hand palmer, left-hand dorsal, left-hand palmer, abdomen, lumbar back, buttocks, perineum, right thigh, left thigh, right lower-leg, left lower-leg, right-foot dorsal, right-foot sole, left-foot dorsal, left-foot sole).
circumstances of burn (9)	injury type (9), source of injury (95), activity (18), category (16), living space (15), locality (24), first-aid received (15), time from injury to admission (7), living circumstances (12)

**Table 2:** Profile of identified clusters by average LOS, TBSA and age.

Cluster (Count)	Characteristics	Average LOS (SD)	Average TBSA (SD)	Average Age
MST_1 (15,418)	No mental based condition	10 (19.42)	6 (7.73)	45 (19.74)
MST_2 (4056)	Presence of mental based condition	20 (32.19)	9 (13.65)	47 (15.01)
SES_1 (9238)	Least deprived (IMD < 20)	11 (19.82)	6 (8.48)	47 (19.83)
SES_2 (8209)	IMD between 20 and 39	12 (22.14)	6 (8.53)	44 (19.00)
SES_3 (2027)	IMD between 40 and 59	12 (22.28)	6 (8.57)	42 (18.3)

## Data Availability

Restrictions apply to the availability of these data. Data were obtained from the International Burn Injury Database (iBID) and applied for via www.ibidb.org, with the permission of iBID governance arrangements (Data citation—iBID: international Burn Injury Database; www.ibidb.org).
